# Pressure and pain In Systemic sclerosis/Scleroderma - an evaluation of a simple intervention (PISCES): randomised controlled trial protocol

**DOI:** 10.1186/1471-2474-13-11

**Published:** 2012-02-06

**Authors:** Begonya Alcacer-Pitarch, Maya H Buch, Janine Gray, Christopher P Denton, Ariane Herrick, Nuria Navarro-Coy, Howard Collier, Lorraine Loughrey, Sue Pavitt, Heidi J Siddle, Jonathan Wright, Philip S Helliwell, Paul Emery, Anthony C Redmond

**Affiliations:** 1 Division of Musculoskeletal Disease, Leeds Institute of Molecular Medicine, University of Leeds, Leeds, UK; 2 Clinical Trials Research Unit, Leeds Institute of Molecular Medicine, University of Leeds, Leeds, UK; 3 Centre for Rheumatology, Hampstead Campus University College London, London, UK; 4 University of Manchester Rheumatic Diseases Centre, Salford Royal Hospitals NHS Trust, Salford, UK; 5 Leeds Institute of Health Sciences, University of Leeds, Leeds, UK; 6 Leeds NIHR Musculoskeletal Biomedical Research Unit, Leeds, UK

**Keywords:** Foot pain, Plantar pressures, Scleroderma, Systemic sclerosis, Insoles

## Abstract

**Background:**

Foot problems associated with Systemic Sclerosis (SSc)/Scleroderma have been reported to be both common and disabling. There are only limited data describing specifically, the mechanical changes occurring in the foot in SSc. A pilot project conducted in preparation for this trial confirmed the previous reports of foot related impairment and reduced foot function in people with SSc and demonstrated a link to mechanical etiologies. To-date there have been no formal studies of interventions directed at the foot problems experienced by people with Systemic Sclerosis. The primary aim of this trial is to evaluate whether foot pain and foot-related health status in people with Systemic Sclerosis can be improved through the provision of a simple pressure-relieving insole.

**Methods:**

The proposed trial is a pragmatic, multicenter, randomised controlled clinical trial following a completed pilot study. In four participating centres, 140 consenting patients with SSc and plantar foot pain will be randomised to receive either a commercially available pressure relieving and thermally insulating insole, or a sham insole with no cushioning or thermal properties. The primary end point is a reduction in pain measured using the Foot Function Index Pain subscale, 12 weeks after the start of intervention. Participants will complete the primary outcome measure (Foot Function Index pain sub-scale) prior to randomisation and at 12 weeks post randomisation. Secondary outcomes include participant reported pain and disability as derived from the Manchester Foot Pain and Disability Questionnaire and plantar pressures with and without the insoles in situ.

**Discussion:**

This trial protocol proposes a rigorous and potentially significant evaluation of a simple and readily provided therapeutic approach which, if effective, could be of a great benefit for this group of patients.

**Trial registration number:**

ISRCTN: ISRCTN02824122

## Background

Systemic sclerosis (SSc)/Scleroderma is a connective tissue disease characterised by excessive collagen production resulting in microvascular and macrovascular damage, fibrosis of the skin and internal organs [1-3]. The prevalence of SSc in the UK is 8.21 per 100,000 [4] with an age of onset as early as the second decade [5]. The young age of the disease onset is reflected in the high societal costs. In 1997 the direct and indirect costs of SSc in the United States was $1.5 billion [6].

Foot problems associated with SSc include: Raynaud's phenomenon, which sometimes can progress to tissue loss/ulceration, subcutaneous calcinosis, skin thickening, callus formation, tendonopathy, foot ulcers, joint space narrowing, bone demineralization, joint subluxation, joint margin erosions and degenerative changes [7-9]. Arthropathy is also common in patients with SSc and is a major determinant of disability [8].

There are parallels between some of the foot symptoms seen in SSc and those in people with rheumatoid arthritis (RA). In RA, inflammatory arthropathy leads to joint damage, which in turn causes increased plantar pressures and altered pressure distribution. Plantar pressure has been reported to be higher under metatarsophalangeal (MTP) joints that are eroded and where fibro-fatty padding displacement has occurred, resulting in values exceeding normal limits [10,11]. Plantar fat-pad atrophy in RA patients also contributes further to altered normal plantar pressure distribution [12,13]. People with SSc have also been shown to suffer from bone erosions [7-9] and subcutaneous fat atrophy [14,15], which could lead to plantar fat-pad atrophy, consequently increasing plantar foot pressures.

Abnormal plantar foot pressures are widely noted as a risk factor for ulceration in people with diabetes and this additive relationship has also been established in diseases closer in nature to SSc, such as RA [16,17]. The consequences of the musculoskeletal changes noted above, on plantar pressures have not been studied in SSc.

In related diseases, high plantar pressures and prolonged duration of loading are associated with pain during walking [11,18]. However, the mechanisms by which foot pain is increased in people with SSc [7,19] have not been investigated. It is well established that pain is common in SSc and is the strongest predictor of physical functioning in SSc patients [20]. In other diseases where foot pain is associated with increased plantar pressure, simple treatments such as cushioned insoles have been shown to be highly effective [21]. Again, the effectiveness of these simple and inexpensive treatments has not been investigated in SSc.

The presence of both abnormal plantar pressures and plantar foot pain in patients with SSc has been confirmed in a pilot study conducted by the investigating team.

### Pilot study

A pilot project [22] investigated 14 people with SSc who provided data on foot pain, impact of pain on foot health status and high resolution plantar pressure data. SSc pain and pressure data were compared with a further 14 healthy controls matched for age and gender.

Patients with SSc reported worse foot pain on a 100 mm visual analogue pain scale (VAPS), SSc mean 36.8 mm (SD = 25.7) compared with a mean of 2.2 mm (SD = 4) in healthy controls. Pressure data demonstrated intense localisation of plantar pressures to discrete areas in close proximity to the MTP joints.

The SSc group had markedly higher forefoot pressures across a range of pressure variables when compared with the control group. Peak plantar pressures and pressure time integrals were particularly high over the first MTP joint.

To address the possible concern that people with SSc have significant systemic complications that may be considered to overwhelm the impact of relatively minor foot complaints, patients were asked explicitly whether reducing foot pain would improve their quality of life. Thirteen out of fourteen respondents indicated explicitly that they would derive benefit from interventions intended to reduce their foot symptoms.

In conclusion, the literature, the pilot study results and the patients themselves indicate that there is a clear need for studies investigating the effect of SSc on plantar pressures and plantar foot pain. Furthermore, if inexpensive and simple interventions known to work in other diseases offer an effective treatment option, then evaluation of these should be a priority for the rheumatology community.

## Methods

### Trial objectives

#### Primary objective

To compare foot pain in participants using a commercially available pressure-relieving insole, to those using the sham insole (control), 12 weeks after randomisation.

#### Secondary objective

To determine the impact of insoles on patient-reported foot disability and function

To assess the impact of the insoles on scleroderma health status

#### Exploratory objective

To explore the relationship between pain, and the distribution and magnitude of plantar pressures (Leeds site only)

To provide further validation data on the newly developed SSc Quality of Life Scale

### Design

Pressure and pain In Systemic Sclerosis/SCleroderma (PISCES) is a pragmatic, phase III, multicentre, randomised, controlled trial.

#### Recruitment and randomisation

Recruitment of the 140 participants will occur over 20 months to allow completion of the twelve-week follow-up within the 24-month time frame of the clinical phase of the study. All potentially suitable patients attending the relevant departments' outpatient's clinics will be approached and provided with a verbal explanation of the trial and a copy of the ethically approved patient information sheet. Arrangements will be made prior to the commencement of the trial to stream potentially suitable patients into early trial clinics. Alternatively, patients identified by other means such as waiting lists or upon review of case records, will receive a letter and the ethically approved patient information sheet from their consultant rheumatologist, which will provide comprehensive information and invite them to participate in the study. Those who express an interest to participate will be invited to contact their local research team to make an appointment to discuss the trial further.

Informed written consent will be obtained from the patients.

Each participant will undergo a pre-trial eligibility screen and baseline assessment prior to randomisation and insole allocation. Randomisation will take place after baseline measures have been recorded to eliminate selection bias

Those participants who meet the eligibility criteria will be randomly allocated to receive one of two interventions. The participants will be randomised on a 1:1 basis to receive either the active intervention (simple pressure-relieving insole) or the control (sham insole). Stratified block randomisation will be used to ensure intervention groups are well-balanced for gender and centre.

Randomisation will be performed centrally using a 24-h, automated randomisation system based at the Leeds Clinical Trials Research Unit,

#### Follow-up

Participants will wear the insoles for 12 weeks and then return for a 12-week follow-up visit prior to exiting the trial. Mid-trial telephone follow-ups will be performed at 6 weeks post randomisation to identify whether the participant has any problems with tolerability, to establish compliance with the intervention and encourage completion of the patient diary. During the 12-week follow-up visit, participants will be asked to complete the same questionnaires as at baseline, supervised by a member of authorised trial staff blinded to insole allocation, and will be required to return their 12-week diary. Participants unable to attend at precisely 12-weeks will be eligible for follow-up between week 11 and week 14. For those participants who cannot attend during this window, questionnaires will be posted to the participants' home address by the participating site. Following the 12-week assessment visit the participants will exit the trial, and any continuation of care will be followed up by the relevant NHS department at the participating centre, as per local policy. A clinical decision as to the appropriateness of providing the participants with the alternative pair of insoles upon trial exit will be made on an individual basis.

In addition, the participants from the Leeds centre will be offered further assessment of bare foot plantar pressures and in-shoe plantar pressures at baseline. Additional written informed consent will be obtained prior to assessment.

**Figure 1:**
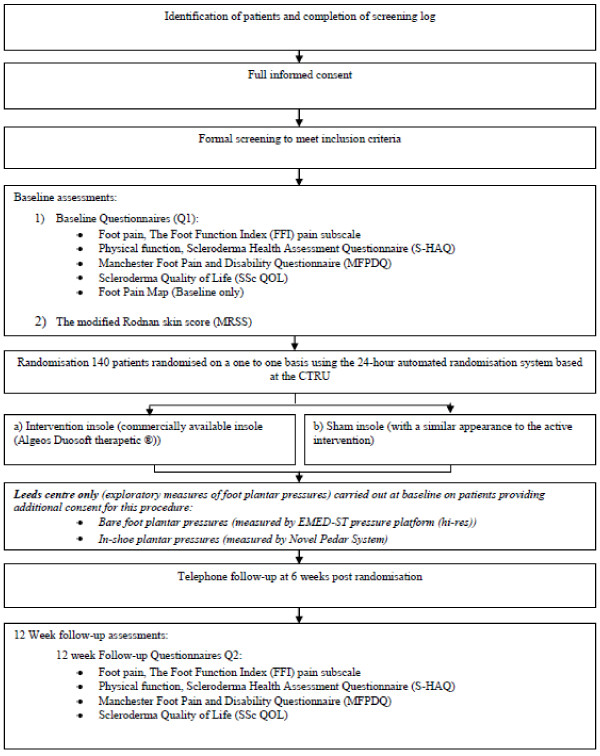
**Trial flow diagram**.

The trial design is summarized in the trial flow diagram (Figure 1)

### Setting & subjects

A total of 140 participants with SSc will be recruited from four centres in the UK which specialise in treatment and study of SSc. Sixty participants will be recruited by the Leeds Teaching Hospitals NHS Trust, Leeds; 25 participants from the Salford Royal Hospitals NHS Trust, Manchester; 40 participants from the Royal Free Hampstead NHS Trust, London; and 15 participants from the Newcastle Upon Tyne Hospitals NHS Foundation Trust, Newcastle.

Potential participants 18 years of age or over will be screened for enrollment. To be included in the trial, participants must meet the following eligibility criteria:

#### Inclusion criteria

1. Consultant diagnosis of SSc or a positive diagnosis of SSc (ARA/ACR 1980 criteria [23]) defined by

Major criterion:

• Proximal diffuse (truncal) sclerosis (skin tightness, thickening, non-pitting induration)

Minor criteria:

• Sclerodactyly (only fingers and/or toes),

• Digital pitting scars or loss of substance of the digital finger pads (pulp loss), and/or

• Bilateral basilar pulmonary fibrosis.

(The patient should fulfill the major criterion or two of the three minor criteria unless a consultant diagnosis has been made).

2. Patient-reported plantar foot pain.

3. Willing and able to comply with the intervention schedule for 12 weeks.

4. Able to provide written informed consent to participate in the study.

#### Exclusion criteria

Patients with the following characteristics are considered ineligible for this study:

1. Disease overlap syndromes (overlap with Inflammatory Arthritis (IA)/RA)

2. History of any lower limb bone or joint orthopaedic surgery within the past 12 months

3. Diagnosis of diabetes

4. Loss of protective sensation on the plantar surface of the foot

5. Current use of prescribed or over-the-counter contoured or made-to-measure insoles/orthoses

6. History of any clinically significant disease or major disorder that in the opinion of the treating clinician or Chief Investigator would not be conducive to trial participation

Details of all eligible potential participants will be recorded, along with detailed reporting of inclusion/exclusion to guard against recruitment bias.

### Intervention

#### Treatment insole

The active intervention to be tested is a commercially available pressure relieving insole (Prothotic™ Duoform, Algeo's Ltd, Merseyside UK, cost £8.55). The insoles are composed of 4 mm thick polyurethane cushioning and a 2 mm Plastazote layer, which provides cushioning and thermal insulation. While a standard insole is being used for consistency, this is a relatively generic product and the insole being evaluated would represent a variety of similar products available on the open market.

#### Sham insole

The sham intervention used in the control arm will consist of a 1 mm thick regenerated leather-board base with a thin (< 1 mm) plastazote cover. The sham intervention will provide a physical insole similar in appearance to the active intervention insole and will look identical once placed in the participants' shoes, but will not offer cushioning or alter the plantar foot pressures. Minimal to none thermal insulating properties are expected, given the thinnest possible plastazote layer achieved in manufacture.

The randomised insole (active or sham) will be fitted into the participants' shoes by the attending clinicians. To standardise the delivery of the intervention (i.e. fitting the insoles) all of the practitioners involved in its delivery will undergo a standardised training programme prior to the start of recruitment.

Participants will be advised to wear the insoles as much as possible during the day for the next 12 weeks. Participants will be provided with a diary providing clear written information regarding the use of, and care of their insoles, including what to do if they become lost or damaged. To monitor intervention compliance and to identify other confounders (e.g. pain and medication), the participants will also be asked to document compliance with insole use in the diary. The diary will also be used to detail the intake of medication specifically for foot pain

#### Blinding

Although the active and sham insoles will look identical when placed in the shoes, it is recognised that protection of blinding may not be possible on all occasions. The following controls will be employed to maintain the blinding of the trial as far as is reasonably practicable:

The sham insole (control) will be similar in appearance to the active insole, and will look identical in appearance once placed in the participants' shoes. The key staff at site involved with the trial will remain blinded to the intervention allocation during the study. This will be achieved by having different teams of therapists responsible for randomising participants to those responsible for administering the assessment questionnaires.

The majority of outcome measures are patient-reported and should not be influenced by clinician knowledge of the insole allocation. The trial participants will be provided with minimal opportunity to meet and discuss interventions with other participants.

### Primary and secondary outcome measures

The primary outcome measure is a reduction in foot pain as measured by the pain subscale of the Foot Function Index (FFI) [24]. The FFI is a validated, self-assessment questionnaire, which was developed to measure the impact of foot pathology in people with RA and which has now been used in a wide range of rheumatic diseases. The pain subscale of the FFI uses visual analogue scales (VAS) to measure the severity of pain using a Likert-type response set with anchors describing "no pain" and "worst pain imaginable", with a mean score derived.

Secondary outcome measures will include participant reported pain and disability as reported via the Manchester Foot Pain and Disability Questionnaire [25], scleroderma function and health status using the Scleroderma Health Assessment Questionnaire (S-HAQ) [26], and quality of life using the newly developed Scleroderma Quality of Life Questionnaire which is an exploratory endpoint due to lack of full validation of this questionnaire to-date.

Objective gait laboratory data will be obtained at the lead centre in Leeds. Plantar foot pressures will be recorded using the Novel EMED-ST (Novel-GmbH, Munich, Germany) pressure platform at baseline to obtain high-resolution measures of pressure and force distributions. Pressures at the shoe/insole interface will also be obtained separately, using the Novel Pedar in-shoe system. The following measures will be reported: maximum mean pressure at forefront of the foot (five metatarsal heads) (kPa), maximum mean pressure at heel of the foot (kPa), contact area at forefront of the foot (cm2), contact area at heel of the foot (cm2), gait velocity (metres per second), cadence (steps per minute) and step/stride length (cm).

### Assessments

Foot Function Index Pain Subscale, S-HAQ, Manchester Foot Pain and Disability Score and Scleroderma QoL will be completed by participants as part of their baseline and 12 week follow-up assessments.

A Foot Pain Map will be completed at baseline to identify the specific area of pain.

The modified Rodnan Skin Score (mRSS) will be calculated for each participant as a measure of disease severity at baseline. The mRSS is a validated measure for dermal skin thickness used universally in rheumatology for this purpose [27,28]. The most widely used version is the 17 site assessment system, which will be employed in this trial and undertaken as part of the baseline assessments.

Participant diaries, instructions on how to complete them and instructions for ongoing use of insoles will be given to the participants prior to them leaving the clinic.

Exploratory measures of foot plantar pressures will be carried out at baseline (after randomisation and insole allocation) on consenting participants at the Leeds centre only. The plantar foot pressure measurements are undertaken in two stages:

Stage 1: Bare foot plantar pressures (measured by EMED-ST pressure platform (hi-res))

Three representative steps, for each foot, are recorded from the participant walking barefoot over the platform using a common 'two-step start' protocol. The EMED platform will allow analysis of a range of pressure, force, area and temporal variables at high resolution (4 sensors/cm2). This will enable a detailed model of the interaction of the participants' foot with the supporting surface to be developed which will inform our characterisation of the baseline effects of the disease. It is not possible however to investigate the effect of the therapy using the platform system, thus requiring a second stage of pressure study.

Stage 2: In-shoe plantar pressures measured by the Pedar System (Novel GmbH, Munich, Germany).

A flexible pressure-measuring insole is inserted into the footwear and the participant undertakes two straight line walks of approximately eight metres, generating approximately 20 representative steps. The trial intervention (active or sham insole) on a separate measure is then added to the footwear, the pressure measuring insole is added over the top, and the process repeated. Randomisation on sequence of measures (with/without insole first) will be employed via a computer generated randomisation.

The in-shoe pressure measurements are derived using a capacitance based matrix of 99 sensors per insole which again generate a range of force, pressure, area and temporal variables characterising the loading of the plantar surface of the foot. Measures are derived automatically within software for pre-determined areas of the foot. After collection but with the participant still present, data are evaluated graphically within the software to ensure the integrity of the measures. Once checked the quantitative data is output to a CSV delimited spreadsheet file for subsequent processing.

### Power calculation/sample size consideration

The trial is powered to detect change in the primary outcome, the Foot Function Index pain subscale. The minimal important difference for FFI pain subscale has been reported to be 12 mm [29]. We have decided to use a conservative minimal important difference between intervention arms of 15 mm.

To detect a difference between groups of 15 mm, with a standard deviation of the response variable = 25.7 (from our pilot data; other published studies providing FFI pain data with insole use have also reported standard deviations of between 20 and 27); alpha = 0.05 and power set at 90%, 63 participants per group are needed. To allow for a 10% drop out rate, a total of 140 participants will be randomised.

### Statistical analysis

The primary outcome of the change in VAS FFI pain subscale between baseline and 12 weeks will be compared between intervention groups using Analysis of Covariance adjusting for baseline pain score, centre and gender. Model assumptions will be checked and if found to be violated data will be transformed prior to analysis or a non-parametric analysis method will be used. Summary statistics of the foot pain score at baseline and week 12 will be presented for the overall pain component as well as each individual pain question. Similar methods will be used for other study questionnaires.

#### Objective gait analysis

All analyses will be according to intention to treat; no interim analyses are planned due to the short follow-up period and the safe nature of the intervention.

## Discussion

The project has been driven from the outset by unmet participant need. First identified as a problem area by participants in the Leeds Connective Tissue Disease Outpatients clinic, the trial was piloted formally with full consultation with people with SSc. While the design of the project has been led methodologically by the CTRU, patient consultation has been undertaken throughout the design phase, and formal patient representation will be undertaken through the trial steering committee.

Although the primary outcome is a patient-reported outcome, the two types of insole (active and sham) are as similar as possible in appearance to conceal the allocation from both patient and clinician and to avoid bias and attrition as far as possible. Randomisation will be performed centrally at the CTRU thus ensuring allocation concealment and minimising any imbalance between the two trial arms.

The trial evaluates a simple intervention carrying minimal risk to participants. There are no invasive tests and no exposure to ionising radiation or other environmental hazards. To maximise participant protection the protocol includes a rescue provision for participants who are unable to tolerate their allocated intervention, or for whom the intervention causes other physical problems.

The 12 weeks follow-up in this trial will allow time for the period of adaptation to the therapy and for the evaluation of its effects. Follow-up in previous studies investigating the effects of orthotic therapy on foot pain varies, but it has been shown that significant decreases in pain can be achieved after 3 months with the therapy [23]. Longer term follow-up might be warranted in future depending on the results of this trial and the impact of any results on future treatment protocols.

This trial will have immediate and direct health benefits for patients with SSc, extending the knowledge of practitioners to effectively manage foot pain in people with SSc. The trial will establish definitively the burden of foot problems in people with SSc and will determine the effectiveness of a simple and inexpensive insole in reducing plantar pressures and ameliorating symptoms.

### Ethical consideration

Ethical and governance approval for this trial has been obtained from the Leeds West Ethics Committee (ref 10/H1307/83) and the Leeds Teaching Hospitals NHS Trust respectively. The trial progress is monitored by an independent Trial Steering Committee.

## Abbreviations

PISCES: Pressure and pain In Systemic Sclerosis/Scleroderma; SSc: Systemic sclerosis; RA: Rheumatoid arthritis; MTP: Metatarsophalangeal; VAPS: Visual analogue pain scale; ARA: American rheumatology association; ACR: American college of rheumatology; NHS: National health service; IA: Inflammatory arthritis; FFI: Foot function index; VAS: Visual analogue scales; S-HAQ: Scleroderma health assessment questionnaire; QoL: Quality of life; mRSS: modified Rodnan Skin Score; CTRU: Clinical trials research unit.

## Competing interests

The authors declare that they have no competing interests.

## Authors' contributions

All authors conceived of the trial and participated in the design. All authors read and approved the final manuscript.

## Pre-publication history

The pre-publication history for this paper can be accessed here:

http://www.biomedcentral.com/1471-2474/13/11/prepub
